# Based on biomedical index data: Risk prediction model for prostate cancer

**DOI:** 10.1097/MD.0000000000025602

**Published:** 2021-04-30

**Authors:** Hanxu Guo, Xianjie Jia, Hao Liu

**Affiliations:** a School of Clinical Medicine, Bengbu Medical College; b Department of Epidemiology and Health Statistics, School of Public Health, Bengbu Medical College; c Department of Pharmacy, Bengbu Medical College, Anhui Biochemical Drug Engineering Technology Research Center, Bengbu, China.

**Keywords:** logistic regression model, neural network model, propensity score matching, prostate cancer, risk factor

## Abstract

To explore the influencing factors of prostate cancer occurrence, set up risk prediction model, require reference for the preliminary diagnosis of clinical doctors, this model searched database through the data of prostate cancer patients and prostate hyperplasia patients National Clinical Medical Science Data Center.

With the help of Stata SE 12.0 and SPSS 25.0 software, the biases between groups were balanced by propensity score matching. Based on the matched data, the relevant factors were further screened by stepwise logistic regression analysis, the key variable and artificial neural network model are established. The prediction accuracy of the model is evaluated by combining the probability of test set with the area under receiver operating characteristic curve (ROC).

After 1:2 PSM, 339 pairs were matched successfully. There are 159 cases in testing groups and 407 cases in training groups. And the regression model was *P* = 1 / (1 + e (0.122 ∗ age + 0.083 ∗ Apo lipoprotein C3 + 0.371 ∗ total prostate specific antigen (tPSA) −0.227 ∗ Apo lipoprotein C2–6.093 ∗ free calcium (iCa) + 0.428 ∗ Apo lipoprotein E-1.246 ∗ triglyceride-1.919 ∗ HDL cholesterol + 0.083 ∗ creatine kinase isoenzyme [CKMB])). The logistic regression model performed very well (ROC, 0.963; 95% confidence interval, 0.951 to 0.978) and artificial neural network model (ROC, 0.983; 95% confidence interval, 0.964 to 0.997). High degree of Apo lipoprotein E (Apo E) (Odds Ratio, [OR], 1.535) in blood test is a risk factor and high triglyceride (TG) (OR, 0.288) is a protective factor.

It takes the biochemical examination of the case as variables to establish a risk prediction model, which can initially reflect the risk of prostate cancer and bring some references for diagnosis and treatment.

## 1 Introduction

### 1.1 Background

Prostate cancer (PCa) is a common malignant tumor of the genitourinary system in elderly men, and its incidence has obvious ethnic and regional differences. Scientific research shows that prostate cancer accounts for the first place in the total number of new cancers in the United States in 2019, and prostate cancer deaths account for 10% of all cancer deaths^[1]^ Prostate cancer screening has always been a controversial health topic. According to the recommendations of the American Urological Association, prostate specific antigen (prostate specific antigen, PSA) screening was generally carried out in the mid-1990s.^[2]^ However, in recent years, many national guidelines have been updated and revised in accordance with the recommendations of U.S. Preventive Services Task Force (USPSTF), which recommended against PSA screening and pointed out that patients may be over diagnosed and over treated.^[3]^ Nowadays PSA has not shown the ability to discriminate clinically important cancers from low-risk tumors. The Prostate Cancer Intervention versus Observation Trial (PIVOT) trial showed no survival benefit from radical prostatectomy in men with PSA ≤ 10 μg/L,^[4]^ which are based on D’Amico criteria as a combination of PSA < 10 μg/L, stage ≤ T2A and Gleason score ≤ 6. It also was reported that more than half of these men underwent unnecessary treatments in Australia.^[5]^ Moreover, PSA screening as currently practiced in the United States provides little to no reduction in prostate cancer mortality or morbidity, does not decrease any-cause mortality, and results in substantial diagnostic and treatment harms and large health-care expenditures. The health importance of prostate cancer and the financial costs to society require improved detection and treatment strategies and more rational use of current options. Until then, men and their health-care providers can make a wise health-care choice by saying no to the PSA test.^[6]^ Prostate cancer screening in China is still in its infancy, but the incidence is increasing rapidly year by year. Some prediction model has been set up, Zhu et al has tested these models that was overestimated by approximately 20% for a wide range of predicted probabilities.^[7]^ Therefore, the construction of prostate cancer risk prediction model for Chinese is an effective and necessary method. There are also many reports about prostate cancer risk prediction in China, but the main factors considered are diagnosis and treatment methods, pathological grading, magnetic resonance imaging, fluorescent probe, etc.^[8,9]^

### 1.2 Objectives

And most of the models do not take into account common clinical indicators such as biochemical examinations. But it is convenient for us to obtain these results from blood test, we can highly increase the efficiency of screening prostate cancer by this innovative model. It may be the first one to use biochemical index to build a prediction model for prostate cancer. In addition, the co-variables of most models will affect the accuracy of the model in varying degrees. Through the innovative method of propensity score matching, this scientific study is expected to better match the case group with the control group, so as to make the co-variables more balanced this model can be used for identifying Chinese who are at a high risk for developing prostate cancer, as well as for cancer screening and developing preventive health strategies.

Artificial neural network, which was first proposed by David and James of San Diego State University. It theoretically proves that any continuity function in a closed interval constantly adjusts the connection strength criterion between neurons in the training of the network. So that the difference between the calculated output dependent variable vector of the network and the dependent variable vector of the known training sample is minimum (that is, the prediction effect is the best).

## 2 Materials and methods

### 2.1 Source of data & sample size

The data of the study were collected from PSA and biochemical examination data of 3000 patients in the National Clinical Medical Science Data Center (301 Hospital), which including 2771 hyperplasia of prostate patients, 112 both prostate hyperplasia and prostate cancer patients and 117 prostate cancer patients. And 112 both prostate hyperplasia and prostate cancer patients recognized as prostate cancer patients with 117 prostate cancer patients are experiment group. 2771 hyperplasia of prostate patients are defined as control group.

### 2.2 Participants & missing data

With the help of Excel 2010 software to sort out the data, we selected a number of biomedical index variables with data, which the missing values are less than 5% of the original data,^[10]^ and this experiment uses SPSS version 25.0 (SPSS Inc. Chicago, IL) software to deal with the missing values of a small number of variables with MCAR method,^[11]^ and extracts relevant variables from the diagnostic information of PSA and biochemical tests.

### 2.3 Predictors

The propensity score matching was achieved on Stata^[12]^ version 12.0 SE (Stata College Station, Texas 77845 USA). In the data after screening by SPSS software, 2771 non-prostate cancer patients were used as the control group, and 229 prostate cancer patients were entered into the Stata database as the experimental group. Based on whether the patient has prostate cancer as a grouping factor, the remaining variables [AGE WEIGHT Body Mass Index Apo lipoprotein A1 (Apo A1) Apo lipoprotein A2 (Apo A2) Apo lipoprotein B2 (Apo B2) Apo lipoprotein C2 (Apo C2) Apo lipoprotein C3 (Apo C3) Apo lipoprotein E (Apo E) albumin alkaline phosphatase lactate dehydrogenase creatine kinase creatine kinase isoenzyme (CKMB) triglyceride high-density lipoprotein cholesterol low-density lipoprotein cholesterol sodium calcium inorganic phosphorus free calcium (iCa) potassium chlorine creatinine (Cre) total prostate specific antigen (tPSA) free prostate specific antigen] are included in the Ps model as covariates. The caliper value is set to 0.05 according to previous scientific research. In this example, it is set to 1:2 matching. After that, the results need to process the validation, which is called the equilibrium Test. If the bias were negative number, it is necessary to process the test again till the matched data fits well.^[13]^

### 2.4 Ethical statement

This study was approved by the Institutional Review Board of the Chinese PLA General Hospital. Participants’ informed consent was waived by the institutional review board because this study involved routinely collected medical data that were anonymously managed in all stages, including the stages of data cleaning and statistical analyses.

### 2.5 Statistical analysis methods

#### 2.5.1 Multivariable logistic regression analysis and artificial neural network

Study was used to set the non-prostate cancer patients in the matching database as the control group and prostate cancer patients as the case group. The case of prostate cancer was assigned a value of 1 and the case of no prostate cancer was assigned a value of 0. Based on the data, literature,^[14–16]^ expert experience and clinical knowledge, the obtained patient information was screened: age, weight, body mass index, Apo A1, Apo A2, Apo B2, Apo C2, Apo C3, Apo E, serum albumin, alkaline phosphatase, lactate dehydrogenase, CKMB, triglyceride, high-density lipoprotein cholesterol and low-density lipoprotein cholesterol. These variables were compared and analyzed for the above-mentioned indicators of these 2 types of patients. Various factors related to prostate cancer were determined using univariable logistic regression analysis methods. Related factors (*P* < .05) were included in multivariable stepwise Logistic. Meanwhile, data with filtered variables were also included in artificial neural network for training and testing. After analysis, meaningful correlation factors were found, and the degree of impact on prostate cancer were based on significant differences.

## 3 Results

### 3.1 Participants

After PSM, the result of the first equilibrium test show that the fitting effects of the variables Na, ALP, and LDLC are not good. As shown in Table 1, the 3 variables are excluded for the second matching. The second equilibrium test shows that all variables have a good fit, such as in Supplementary Table 1, 339 pairs (228 PCa cancer patients regarding as the experimental groups and 456 non-PCa regarding as the control groups were successfully matched, of which 2 groups were unsuccessfully matched, and the variables in the successfully matched experimental group and control group were consistent with normal distribution. A flow chart is provided in Supplement Figure 2.

**Table 1:** Basic demographics.

	Before PSM			Optimization degree (%)	After PSM			Optimization degree (%)	
Variable	Non-PCa n = 458	PCa n = 229	*P* value	Relative bias	Non-PCa n = 458	PCa n = 229	*P* value	Relative bias	Bias (%)
AGE	54.733	66.279	.000	119.3	66.899	66.274	.504	−6.5	94.6
WEIGHT	77.019	72.611	.000	−41.5	72.367	72.686	.758	3.0	92.8
BMI	25.882	24.876	.000	−31.9	24.901	24.906	.987	0.2	99.5
Apo C3	11.801	10.956	.109	−8.8	10.343	10.975	.484	6.6	25.3
Apo A2	27.925	26.724	.001	−23.9	26.674	26.761	.851	1.7	92.8
Apo C2	5.1609	4.0804	.000	−37.8	4.0751	4.0728	.847	−0.1	99.8
Apo E	4.2864	4.7976	.000	30.1	4.9889	4.8034	.365	−10.9	63.7
ALB	43.553	41.414	.000	−54.7	41.168	41.395	.642	5.8	89.4
CKMB	12.48	16.034	.000	36.7	16.548	16.095	.682	−0.3	87.3
fPSA	0.4132	3.9925	.000	14.8	1.1049	1.538	.136	1.8	87.9
tPSA	1.9376	25.824	.000	31.8	8.9587	14.662	.061	5.8	76.1
Ca	2.2898	2.2544	.000	−32.1	2.2439	2.2541	.387	9.2	71.2
CL	105.05	103.22	.000	−58.5	102.93	103.23	.341	9.6	83.7
IP	1.1793	1.1541	.064	−13.2	1.1378	1.155	.340	9.1	31.5
iCa	1.2019	1.1589	.000	−73.8	1.1521	1.1586	.217	11.2	84.8
LDH	150.65	158	.000	22.3	159.28	158.13	.722	−3.5	84.3
CK	103.83	98.169	.443	−6.4	101.64	98.779	.670	−3.2	49.5
Cre	80.962	84.258	.318	6.8	85.771	84.32	.722	−3.0	56.0
TG	1.9869	1.4027	.000	−42.2	1.411	1.4054	.330	−0.4	99.0
HDL-C	1.1229	1.217	.000	31.2	1.2167	1.223	.839	−2.1	93.4
Apo A1	1.1679	1.3294	.000	67.0	1.3451	1.3301	.566	−6.2	90.7
K	4.0508	4.0311	.409	−6.1	4.0239	4.0322	.799	2.6	58.0
Apo B	0.8912	0.96598	.000	31.8	0.99848	0.96743	.218	−13.2	58.5
Na	141.9	141.94	.851	1.2	141.79	141.91	.661	4.7	−300.7
ALP	68.444	71.784	.039	9.1	66.53	71.433	.159	13.3	−46.8
LDL-C	2.8569	2.9132	.322	6.8	2.919	3.0009	.330	−10.0	−45.5

### 3.2 Model specification

Matched data were entered into univariable logistic regression to screen variables. And pick up which one has statistical significance (*P* < .05). The analysis results are shown in Supplementary Table 2. As for PSA and calcium parameter, only one of each type has been selected. The above table shows that the total PSA is greater than the free PSA in the Wald test. And in previous the clinical analysis,^[17,18]^ total PSA always reflects the serum antigen value, so tPSA was included in the model and free PSA was excluded. But based on numbers of studies,^[18,19]^ iCa was included in the model, which has not been included in prediction model before.^[20]^ In summary, the variables included in the model at this time are: age, weight, height, body mass index, Apo A1, Apo A2, Apo C2, Apo C3, Apo E, serum albumin, Cre, CKMB, triglyceride, high-density lipoprotein cholesterol, iCa, tPSA. Multivariable stepwise logistic regression analysis was performed in Table 2. Result analysis: Of the 15 related factors of prostate cancer, there were 9 related factors with statistically significant changes in goodness of fit (step 9): age, Apo C2, Apo C3, Apo E, CKMB, triglycerides, high-density lipoprotein cholesterol, iCa, tPSA.

**Table 2:** Multivariable regression analysis.

Step	Relative variable	β	S.E	Wald	*P*	OR
Step 1	AGE	0.168	0.014	152.430	.000	1.183
	constant	−10.338	0.824	157.255	.000	0.000
Step 2	AGE	0.161	0.014	130.246	.000	1.174
	iCa	−11.848	2.025	34.238	.000	0.000
	constant	4.139	2.467	2.816	.093	62.758
Step 3	AGE	0.132	0.015	78.299	.000	1.141
	iCa	−13.053	2.160	36.536	.000	0.000
	TG	−0.593	0.126	22.060	.000	0.553
	constant	8.492	2.716	9.774	.002	4877.526
Step 4	AGE	0.142	0.016	81.421	.000	1.153
	iCa	−13.595	2.253	36.418	.000	0.000
	HDLC	−2.209	0.450	24.129	.000	0.110
	TG	−0.882	0.151	33.932	.000	0.414
	constant	11.697	2.880	16.493	.000	120193.950
Step 5	AGE	0.137	0.016	72.925	.000	1.147
	iCa	−13.295	2.323	32.764	.000	0.000
	HDLC	−2.679	0.481	31.005	.000	0.069
	TG	−1.328	0.211	39.586	.000	0.265
	Apo E	0.349	0.102	11.776	.001	1.418
	constant	11.310	2.977	14.432	.000	81646.190
Step 6	AGE	0.132	0.016	67.985	.000	1.141
	iCa	−13.338	2.373	31.592	.000	0.000
	CKMB	0.080	0.029	7.537	.006	1.083
	HDLC	−2.652	0.497	28.505	.000	0.070
	TG	−1.321	0.217	36.990	.000	0.267
	Apo E	0.344	0.101	11.531	.001	1.410
	constant	10.493	3.007	12.179	.000	36072.605
Step 7	AGE	0.111	0.017	40.784	.000	1.117
	iCa	−13.081	2.674	23.926	.000	0.000
	CKMB	0.085	0.032	7.327	.007	1.089
	tpsa	0.352	0.066	28.124	.000	1.422
	HDLC	−2.138	0.563	14.404	.000	0.118
	TG	−1.294	0.250	26.736	.000	0.274
	Apo E	0.408	0.106	14.836	.000	1.504
	constant	9.310	3.361	7.670	.006	11042.987
Step 8	AGE	0.112	0.018	40.924	.000	1.119
	Apo C3	0.062	0.026	5.549	.018	1.064
	iCa	−13.831	2.741	25.457	.000	0.000
	CKMB	0.083	0.032	6.834	.009	1.086
	tpsa	0.363	0.069	27.811	.000	1.438
	HDLC	−2.432	0.589	17.071	.000	0.088
	TG	−1.687	0.310	29.538	.000	0.185
	Apo E	0.387	0.112	11.907	.001	1.472
	constant	10.484	3.455	9.210	.002	35751.766
Step 9	AGE	0.108	0.018	38.036	.000	1.114
	Apo C2	−0.211	0.100	4.441	.035	0.810
	Apo C3	0.089	0.035	6.544	.011	1.093
	iCa	−13.158	2.755	22.810	.000	0.000
	CKMB	0.078	0.031	6.234	.013	1.082
	tpsa	0.368	0.071	27.160	.000	1.445
	HDLC	−2.269	0.596	14.521	.000	0.103
	TG	−1.405	0.324	18.847	.000	0.245
	Apo E	0.412	0.108	14.596	.000	1.510
	constant	9.927	3.470	8.183	.004	20466.604

### 3.3 Model performance

The Logistic regression equation was obtained as follows: *P* = 1/(1 + e (0.122 ∗ age + 0.083 ∗ Apo C3 + 0.371 ∗ tPSA total − 0.227 ∗ Apo C2 − 6.093 ∗ iCa + 0.428 ∗ Apo E − 1.246 ∗ triglyceride − 1.919 ∗ high density lipoprotein cholesterol + 0.083 ∗ CKMB).

**Figure 1:**
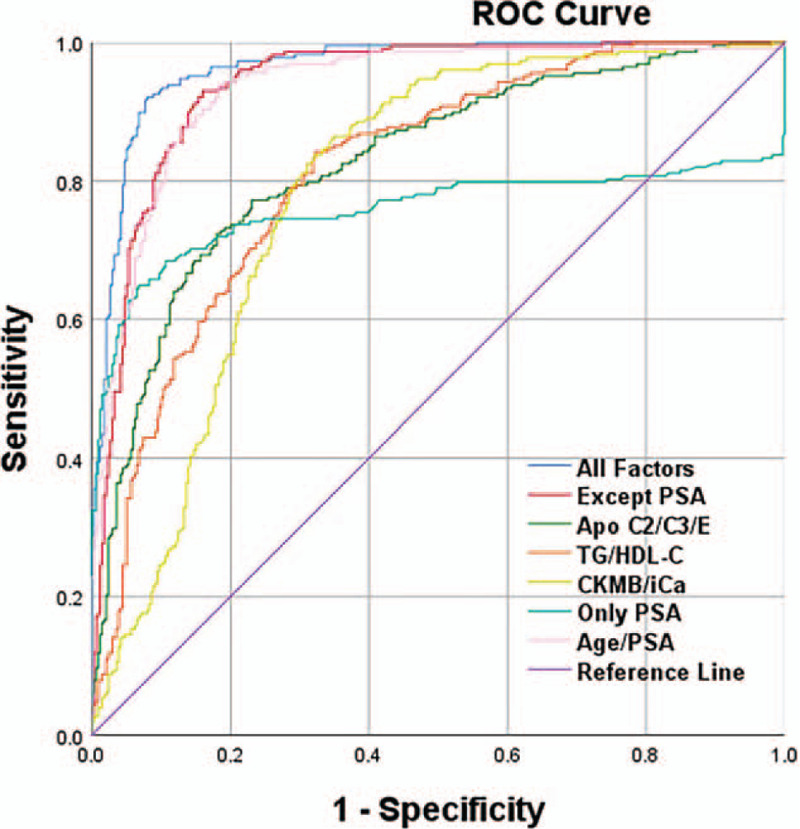
Comparison of ROC curves. ROC curves showed that the new model that based on the multivariable logistic regression has higher prediction efficiency than the model that only be built on PSA.

The new model was compared with the simulated probability of total diagnosis of prostate cancer only by PSA, and the diagnostic efficiency was judged according to the ROC curve (Fig. 1). It can be seen that the prediction efficiency of the new model (measure of area: 0.963) is significantly higher than that of only PSA (measure of area: 0.785) as a single factor.

**Figure 2:**
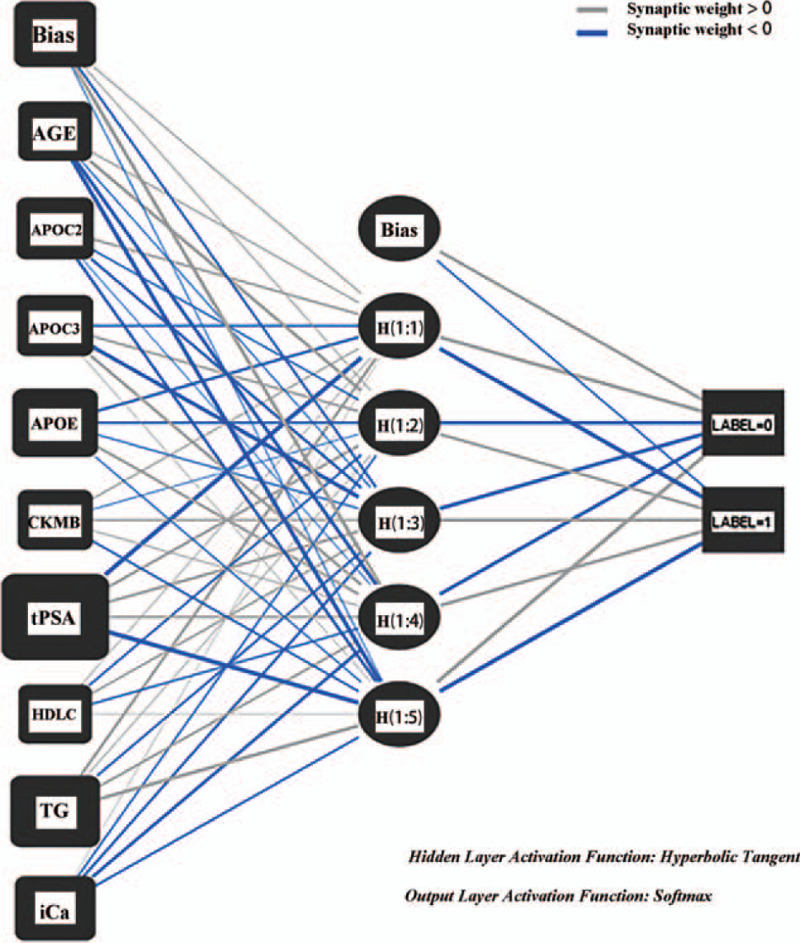
Artificial neural network. Artificial neural network shows that the different importance of independent variables, which similar to the model that based on logistic regression.

Data with 9 related factors were resulted from univariable logistic regressions were put into artificial neural network model for training and testing. The results are shown in Figure 2.

In terms of model specification, artificial neural networks require no knowledge of the data source but, since they often contain many weights that must be estimated, they require large training sets.^[21,22]^ The system will randomly select 71.9% of the cases as the training set for modeling and 28.1% of the cases as the test set to test the quality of the model.^[23]^ There is no significant difference between the verification model and the test model (94.8%, respectively, 91.8%), so there is no overtraining in the model, and the importance of each variable is similar to that in the logistic model

At the same time, compare the logistic regression model with the artificial neural network model to simulate the diagnosis efficiency, and judge the diagnosis efficiency according to the ROC curve, as shown in Supplementary Figure 1.

The diagnosis efficiency of neural network model (ROC, 0.963; 95% confidence interval, 0.951–0.978) is slightly higher than that of logistic regression model (ROC, 0.983; 95% confidence interval, 0.964–0.997) (Supplemental Table 3).

## 4 Discussion

With more debate on the accuracy of the PSA screening on prostate cancer, a large number of researchers found that PSA screening may lead to overtreatments and overdiagnoses. This concern driven the process of not only new diagnostic and prognostic tools but also models to predict the risk of prostate cancer. Based on the fundamental realities of the country, this study innovatively evaluated several biomedical risk factors suspected to have a role in predisposing men to prostate cancer and determined some significant variables to develop a risk prediction model of prostate cancer in China.

The findings of this study will be helpful in deciding on future health policies and preventive strategies for prostate cancer in China. This study is the first to develop a risk prediction model of prostate cancer, based on biomedical information. With the 96.3% diagnostic efficiency of logistic regression model and 98.3% that of neural network model, our model is an excellent discriminator, compared with former models, including those that combine PSA values with PSA relatives and prostate volume.

Former prediction models for prostate cancer have been reported.^[24,25]^ Most of previous models have concentrated on the PSA test for prostate cancer screening and ignore that the cut-off values of specificity and sensitivity are indistinct. High predictive accuracy and discrimination is completed in several models, for instance, Prostaclass I (AUC, 0.79), Chun (AUC, 0.76), Karakiewcz (AUC, 0.74), and Finne (AUC, 0.74).^[26]^ But they still limited in high probability of over diagnosis and overtreatment.^[27]^ In the case of the absence of PSA screening in China, multiple other additive parameters such as MRI, DRE, and prostate volume were added to increase the predictive accuracy of PSA testing in the developmental prediction model.

This study not only adds the PSA also include multiple biomedical parameters, which are easily obtained in Chinese blood test report. Relevant reports are few, but risk factors above are demonstrated in experiments that are explored by researchers.

### 4.1 Interpretation

#### 4.1.1 Apo lipoprotein E, Apo lipoprotein C2, and Apo lipoprotein C3

Despite recognized risk factors such as age and PSA,^[28,29]^ which have also been demonstrated in the experiment, some risk factors that are still controversial also be found in this experiment. Apo lipoprotein E is also an important cholesterol regulatory protein. The main genetic subtypes of Apo lipoprotein E in the body are E3/E3, E3/E4, E2/E3, E2/E2, and E4/E4. The Apo lipoprotein E is also an important cholesterol regulatory protein. At present, studies at home and abroad have shown that the relationship between the invasion and Gleason score of prostate cancer cells and their genotypes in vivo is controversial. In an earlier study, Liu et al, through a case-control study,^[30]^ indicated that the E4 genotype and its allele were not associated with the pathogenesis and prognosis of prostate cancer, but could not explain the experiment conducted by Ifere et al^[31]^ to prove that Apo lipoprotein E2/E4 is a risk factor for prostate cancer. In recent years, Yencilek et al^[32]^ believe that the presence of E4 may reduce the possibility of prostate cancer, but still believe that E3/E3 is a major risk factor for prostate cancer and affecting Gleason scores. Recently, a research by Asare et al,^[33]^ had proved Apo E could potentially be a discriminating biomarker for prostate cancer. Our study has supported this opinion.

Logistic stepwise analysis showed that Apo lipoprotein E was a risk factor for prostate cancer, with an OR of 1.535. From the side to verify the relationship between Apo lipoprotein E and prostate cancer, in clinical work, can guide patients to do some genetic tests, in order to better diagnose and guide the next treatment.

Besides, in the past, there may be not experiments that have stated Apo lipoprotein C2 and Apo lipoprotein C3 are associated with prostate cancer. This model probably the first 1 to demonstrated that Apo lipoprotein C2 is the protective factor and Apo lipoprotein C3 is the risk factor of prostate cancer, with OR values of 0.797 and 1.086, respectively. Its internal mechanism we guess may be that tumor patients accelerate the decomposition of en-dogenous lipids and the transformation and oxidation of free fatty acids and glycerol due to the invasion of tumor tissue and the increase of the level of lipid metabolic hormones and insulin tolerance in the host.

#### 4.1.2 Triglyceride and high density lipoprotein cholesterol

TG provides essential fatty acids in lipid metabolism, which still remains controversy among scholars. Allot et al^[34]^ believe that the increase of serum triglycerides is related to the occurrence of prostate cancer. However, Asare et al^[33]^ have not found any significance difference with TG between Benign prostatic hyperplasia and prostate cancer. High density lipoprotein cholesterol (HDL-C) is an anti-atherosclerotic lipoprotein that transfers cholesterol from extra hepatic tissue to liver for metabolism. A case-control study conducted by Magura et al showed that^[35]^ High TC (total cholesterol), high LDL-C (low density lipoprotein cholesterol), and low HDL-C may be risk factors for prostate cancer. However, the discussion on the relationship between blood lipids and prostate cancer is still controversial. In general, No experiments based on Chinese people have been created in order to study on the internal association between TG, HDL-C and prostate cancer.

Consistent with the results of this study, triglyceride and high density lipoprotein cholesterol were protective factors for prostate cancer, with OR values of 0.288 and 0.147, respectively, reflecting that low triglyceride and low high density lipoprotein cholesterol increase the risk of prostate cancer.

#### 4.1.3 Free calcium

Calcium ion is an indispensable ion for maintaining normal physiological activities of the body, and it is very important for the regulation of electrical activity on both sides of the cell membrane. At the same time, calcium intake can affect the signal transduction pathway, promote the secretion of vascular endothelial factor and increase hypoxia inducible factor. In the last century, X-ray microanalysis has been performed on freeze-dried cryosections of normal, hyperplastic, and neoplastic human prostate, studies had found that calcium is the major prostate acinar cell cation.^[36]^ In recent years, a number of studies at home and abroad have also shown that calcium-binding proteins can activate a variety of pathways to promote the spread of invasive prostate cancer cells.^[37,38]^ In addition, our experiment results, which have shown, high iCa may avoid calcium-binding proteins creating.

In this experiment, as a protective factor of prostate cancer, the OR value of iCa is 0.002, which is of little statistical significance and has little guiding significance for clinical work, but it has a certain enlightening effect on scientific research.

#### 4.1.4 Creatine kinase isoenzyme

Creatine kinase isoenzyme (CK-MB) is mainly used in the diagnosis of myocardial infarction. However, in the early years, A Gries et al^[39]^ accidentally found that the number of CK-MB may be related to prostate tumors. Since then, based on the continuous development of proteomics, many scholars^[40,41]^ have suggested that CK-MB as a marker of malignant tumor should be included in clinical screening. Up to 2015, there is no systematic review or clinical application report on the false increase of CK activity caused by other CK-MB isozymes in malignant tumors.^[42]^

In this study, CKMB is a risk factor for prostate cancer, the OR value is 1.086, the increase of CKMB will increase the risk of prostate cancer. This suggests that researchers should study and develop new indicators about CK-MB, and provide evidence for previous experiments.

### 4.2 Implications

Clinically, researchers should also pay attention to patients’ cardiovascular disease and make a timely distinction from prostate cancer. Furthermore, latest research indicates that has-miR-940 act as a diagnostic and prognostic tool for prostate cancer.^[43]^ Besides, ix co-expressed miRNAs (hsa-miR-17-3p, −377-3p, −410-3p and −495) and p2 miRNA panel (hsa-miR −377-3p, −410-3p, −27a-3p, 149-5p and 940) mainly associated with prostate cancer.^[44]^ In other aspects, respect is a non-invasive, label-free, laser-based technique that identifies molecular composition of tissues and cells, which experiments have demonstrated that such technique could provide insight into different pathways leading to pre-cancerous anal squamous intraepithelial lesions.^[45]^ It is believed that can also be extended in several carcinoma, including prostate cancer. Meanwhile, previous studies had indicated that bone scan-negative patients with a relatively high PSA level and velocity, the risk of distant disease is much greater, and PET imaging^[46]^ may serve as a useful whole-body staging method.^[47]^ Now more tracers for PET/CT are shown to be more accurate in the detection of recurrent disease as compared with radiolabelled choline PET/CT.^[48]^ It is exciting for the clinical doctors to improve the efficiency of diagnostic tools in the future. In the level of genomics, researchers suggested that variations in tumor epigenetic landscape of individuals are partly mediated by genetic differences, which may affect prostate cancer progression.^[49]^ It inspires us that these results could be applied in clinical practice that is helpful to distinguish indolent prostate cancer from advanced disease.

## 5 Conclusion

In this study, the innovative use of propensity score matching method reduces the differences between groups in the data, makes a better comparison between groups. In addition, this experiment also introduces the neural network model to improve the adaptability of the model to the nonlinear relationship between different complex variables. Among them The logistic regression model performed very well (ROC, 0.963; 95% confidence interval, 0.951–0.978) and artificial neural network model (ROC, 0.983; 95% confidence interval, 0.964–0.997) The most important was that Apo lipoprotein E, Apo lipoprotein C2, Apo lipoprotein C3, Triglyceride, High density lipoprotein cholesterol iCa and CKMB , are risk factors related to prostate cancer that have never been discovered or disputed, increase the trust of the known evidence or point out the direction for future research. What is more, increasing the apo test in the physical routine examination is a better way to improve the accuracy of the prostate cancer screening.

### 5.1 Limitation

This study also has many shortcomings: the final model does not involve pathological diagnosis, MRI imaging, Gleason score, digital rectal examination and other strong pathological factors as risk factors, since all data were collected through routine physical examinations; the short periods between risk measure and incidence of prostate cancer identification; and the exclusion of additional unmeasured or unexamined variables. Besides, the number of patients need to be increased and expected to do conduct a multi-center study. In addition, there are not many related research reports in China, and the experiments based on a certain factor are not convincing, and more experts and scholars are needed to provide external medical record data to verify the advantages and disadvantages of the model. For propensity score matching, Propensity score matching cannot assess and balance all possible outcome-influencing factors,^[50]^ such as the LDL, several researches have indicated the pathways that are activated by LDL.^[51]^ But the LDL has been excluded in this model. Furthermore, the availability of clinical practices based on a large number of validations to test.^[52]^ Despite these limitations, this is the first significant study of clinical prediction modeling assessing the incidence risk of prostate cancer by biomedical parameters in China. Besides, these new parameters that were digged in this study also inspire us to explore the inner connection and molecular functions between the biomedical index and prostate cancer. In order to better apply this model and related research to China's domestic clinical work. Up to now, there are a lot of risk calculators have been set up,^[53]^ we hope more and more scholars to work in this area.

## Acknowledgments

Thanks to the Population and Health Science Data Sharing Platform, National Clinical Medical Science Data Center 301 Hospital provides data support and related help for this article. Thanks for M.D. Xue Sheng's careful guidance.

## Author contributions

**Conceptualization:** Hanxu Guo, Xianjie Jia.

**Data curation:** Hanxu Guo.

**Formal analysis:** Hanxu Guo.

**Funding acquisition:** Hanxu Guo.

**Investigation:** Hanxu Guo.

**Methodology:** Hanxu Guo.

**Project administration:** Hanxu Guo.

**Resources:** Hanxu Guo.

**Software:** Hanxu Guo.

**Supervision:** Hanxu Guo.

**Validation:** Hanxu Guo.

**Visualization:** Hanxu Guo.

**Writing – original draft:** Hanxu Guo.

**Writing – review & editing:** Hao Liu, Xianjie Jia.

## Supplementary Material

Supplemental Digital Content

## Supplementary Material

Supplemental Digital Content

## Supplementary Material

Supplemental Digital Content

## Supplementary Material

Supplemental Digital Content

## Supplementary Material

Supplemental Digital Content
