# Supplementary material for: Based on biomedical index data: Risk prediction model for prostate cancer
Source: Medicine (Baltimore). 2021 Apr 30;100(17):e25602. doi: 10.1097/MD.0000000000025602 (PMC8084031; doi:10.1097/MD.0000000000025602)
Supplement: Supplemental Digital Content [file medi-100-e25602-s001.doc]

| Variable | Before Matching | | | Optimization degree (%) | After Matching | | | Optimization degree (%) | Bias(%) |
| --- | --- | --- | --- | --- | --- | --- | --- | --- | --- |
|  | Non-PCa  n=458 | PCa  n=229 | P Value | Relative bias | Non-PCa  n=458 | PCa  n=229 | P Value | Relative bias |  |
| AGE | 54.733 | 66.279 | 0.000 | 119.3 | 67.216 | 66.238 | 0.289 | -10.1 | 91.5 |
| WEIGHT | 77.019 | 72.611 | 0.000 | -41.5 | 73.269 | 72.674 | 0.546 | -5.6 | 86.5 |
| BMI | 25.882 | 24.876 | 0.000 | -31.9 | 25.156 | 24.904 | 0.375 | 0.2 | 74.9 |
| Apo CⅢ | 11.801 | 10.956 | 0.109 | -8.8 | 10.521 | 10.957 | 0.629 | 4.5 | 48.4 |
| Apo A2 | 27.925 | 26.724 | 0.001 | -23.9 | 26.539 | 26.742 | 0.663 | 4.0 | 83.1 |
| Apo CⅡ | 5.1609 | 4.0804 | 0.000 | -37.8 | 4.1093 | 4.0678 | 0.847 | -1.5 | 96.2 |
| Apo E | 4.2864 | 4.7976 | 0.000 | 30.1 | 5.0204 | 4.7961 | 0.292 | -13.2 | 56.1 |
| ALB | 43.553 | 41.414 | 0.000 | -54.7 | 40.618 | 41.411 | 0.124 | 20.3 | 62.9 |
| CKMB | 12.48 | 16.034 | 0.000 | 36.7 | 16.089 | 16.056 | 0.975 | -0.3 | 99.1 |
| fPSA | 0.41327 | 3.9925 | 0.000 | 14.8 | 1.1743 | 1.6413 | 0.144 | 1.9 | 87.0 |
| tPSA | 1.9376 | 25.824 | 0.000 | 24.4 | 10.757 | 15.919 | 0.146 | 5.3 | 78.4 |
| Ca | 2.2898 | 2.2544 | 0.000 | -32.1 | 2.238 | 2.2546 | 0.190 | 15.0 | 53.2 |
| CL | 105.05 | 103.22 | 0.000 | -58.5 | 102.99 | 103.24 | 0.443 | 8.0 | 86.3 |
| IP | 1.1793 | 1.1541 | 0.064 | -13.2 | 1.1346 | 1.1544 | 0.300 | 10.4 | 21.7 |
| iCa | 1.2019 | 1.1589 | 0.000 | -73.8 | 1.1505 | 1.1585 | 0.141 | 13.9 | 81.2 |
| LDH | 150.65 | 158 | 0.000 | 22.3 | 159.42 | 157.97 | 0.722 | -4.4 | 80.2 |
| CK | 103.83 | 98.169 | 0.443 | -6.4 | 102.02 | 98.57 | 0.624 | -3.9 | 39.1 |
| Cre | 80.962 | 84.258 | 0.318 | 6.8 | 86.511 | 84.36 | 0.633 | -4.4 | 34.8 |
| TG | 1.9869 | 1.4027 | 0.000 | -42.2 | 1.4103 | 1.4059 | 0.950 | -0.3 | 99.2 |
| HDL-C | 1.1229 | 1.217 | 0.000 | 31.2 | 1.2018 | 1.2147 | 0.658 | 4.3 | 86.3 |
| Apo A1 | 1.1679 | 1.3294 | 0.000 | 67.0 | 1.3315 | 1.3283 | 0.903 | -1.3 | 98.0 |
| K | 4.0508 | 4.0311 | 0.409 | -6.1 | 4.0384 | 4.0343 | 0.901 | -1.3 | 79.1 |
| Apo B | 0.8912 | 0.96598 | 0.000 | 31.8 | 0.99646 | 0.96687 | 0.239 | -12.6 | 60.4 |

**Supplementary Table 1. The second univariate analysis before and after PSM**

Values are presented as mean; PSM: propensity score matching.
