# Supplementary material for: Based on biomedical index data: Risk prediction model for prostate cancer
Source: Medicine (Baltimore). 2021 Apr 30;100(17):e25602. doi: 10.1097/MD.0000000000025602 (PMC8084031; doi:10.1097/MD.0000000000025602)
Supplement: Supplemental Digital Content [file medi-100-e25602-s002.docx]

**Supplementary Figure 2. Flow Chart**

**The flow chart showed that the complete process of the Method Section.**

**

**

**Footnote**

Relevant abbreviations has been supplied as the Supplemental Table 3 and can be read in the Page 2. Prof. Jia Xianjie and Ph.D. Liu Hao are both corresponding authors.
