# Supplementary material for: Based on biomedical index data: Risk prediction model for prostate cancer
Source: Medicine (Baltimore). 2021 Apr 30;100(17):e25602. doi: 10.1097/MD.0000000000025602 (PMC8084031; doi:10.1097/MD.0000000000025602)
Supplement: Supplemental Digital Content [file medi-100-e25602-s003.doc]

**Supplementary Table 2 Single factor Logistic regression results of prostate cancer**

| Relative Variable | | β | S.E | Wals. | P value |
| --- | --- | --- | --- | --- | --- |
| AGE | 0.168 | | 0.014 | 152.495 | <0.05 |
| WEIGHT | -0.047 | | 0.008 | 31.953 | <0.05 |
| BMI | -0.138 | | 0.028 | 23.965 | <0.05 |
| APOA2 | -0.140 | | 0.019 | 56.304 | <0.05 |
| APOC2 | -0.422 | | 0.044 | 90.271 | <0.05 |
| Apo CⅢ | -0.109 | | 0.016 | 47.670 | <0.05 |
| APOE | -0.147 | | 0.043 | 11.494 | <0.05 |
| ALB | -0.301 | | 0.032 | 89.743 | <0.05 |
| ALP | 0.002 | | 0.003 | 0.753 | 0.386 |
| CKMB | 0.118 | | 0.021 | 31.263 | <0.05 |
| fPSA | 1.602 | | 0.244 | 43.081 | <0.05 |
| tPSA | 0.476 | | 0.053 | 79.872 | <0.05 |
| Na | 0.028 | | 0.035 | 0.657 | 0.418 |
| Ca | -6.962 | | 0.959 | 52.755 | <0.05 |
| CL | -0.153 | | 0.028 | 29.301 | <0.05 |
| IP | -0.722 | | 0.494 | 2.133 | 0.144 |
| ica | -14.179 | | 1.676 | 71.617 | <0.05 |
| LDH | 0.008 | | 0.003 | 7.835 | 0.005 |
| CK | 0.000 | | 0.002 | 0.017 | 0.896 |
| Cre | 0.011 | | 0.005 | 4.300 | 0.038 |
| TG | -0.997 | | 0.117 | 72.179 | <0.05 |
| HDL-C | 0.601 | | 0.255 | 5.546 | 0.019 |
| LDL-C | -0.165 | | 0.095 | 3.034 | 0.082 |
| APOA1 | 1.861 | | 0.355 | 27.547 | <0.05 |
| APOB | 0.159 | | 0.345 | 0.212 | 0.645 |
| K | -0.714 | | 0.260 | 7.555 | 0.006 |
