# Supplementary material for: Based on biomedical index data: Risk prediction model for prostate cancer
Source: Medicine (Baltimore). 2021 Apr 30;100(17):e25602. doi: 10.1097/MD.0000000000025602 (PMC8084031; doi:10.1097/MD.0000000000025602)
Supplement: Supplemental Digital Content [file medi-100-e25602-s004.docx]

**Supplementary Figure 1. The ROC curve of BP Neural Network and Multivariable Logistic Regression Model Comparison**

**ROC curves showed that the BP Artificial neural network model has higher prediction efficiency than the model that based on multivariable logistic regression**

**
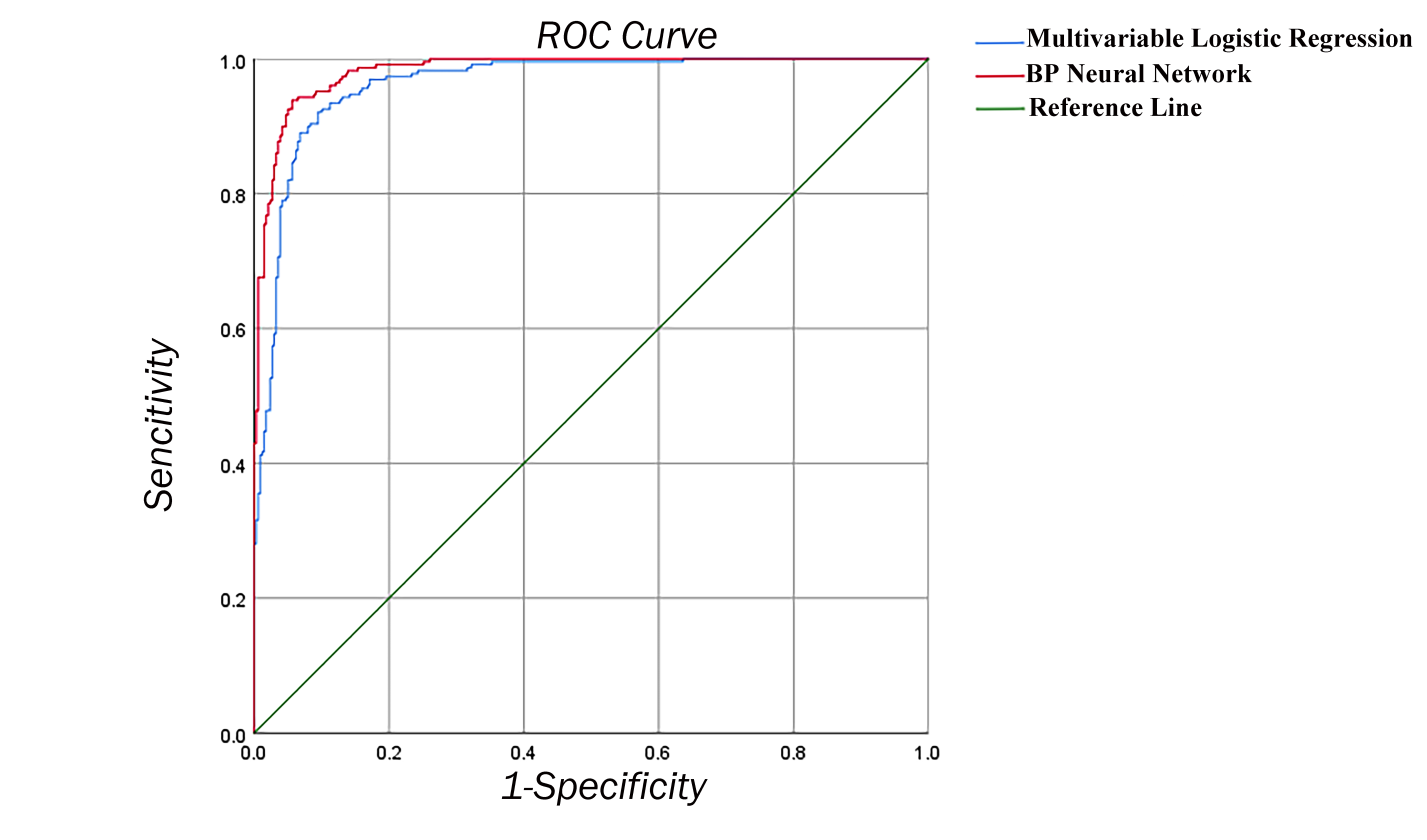
**
