# Supplementary material for: Based on biomedical index data: Risk prediction model for prostate cancer
Source: Medicine (Baltimore). 2021 Apr 30;100(17):e25602. doi: 10.1097/MD.0000000000025602 (PMC8084031; doi:10.1097/MD.0000000000025602)
Supplement: Supplemental Digital Content [file medi-100-e25602-s005.doc]

**Supplementary Table 4. ROC Area**

| Variable | Area | S.E. | 95%C.I. | |
| --- | --- | --- | --- | --- |
|  |  |  | Lower | Upper |
| All Factors | 0.963 | 0.007 | 0.951 | 0.978 |
| Except PSA | 0.94 | 0.01 | 0.92 | 0.959 |
| Apo C2/C3/E | 0.829 | 0.018 | 0.794 | 0.864 |
| TG/HDL-C | 0.816 | 0.018 | 0.782 | 0.851 |
| CKMB/iCa | 0.791 | 0.019 | 0.754 | 0.828 |
| Only PSA | 0.785 | 0.026 | 0.708 | 0.808 |
| Age/PSA | 0.934 | 0.011 | 0.913 | 0.955 |

**S.E: Standard Error , 95%C.I.: Confidence Interval.**
